# Older people in the world of esport: a qualitative study

**DOI:** 10.3389/fpsyg.2024.1460966

**Published:** 2024-10-18

**Authors:** Catherine Esnard, Marion Haza, Rebeca Grangeiro

**Affiliations:** ^1^Centre de Recherches sur la Cognition et l’Apprentissage, Département de Psychologie, Université de Poitiers, Poitiers, France; ^2^Laboratoire de Psychologie Clinique, Psychopathologie, Psychanalyse, Université Paris Cité, Paris, France

**Keywords:** older people, esport, psychological well-being, qualitative study, social psychology

## Abstract

**Introduction:**

The inclusion of older players in the gaming world is growing rapidly, leading to the emergence of specific categories for them at major esport events. The aim of this study is to analyze the psychological issues faced by older people when engaging with the competitive context of esport.

**Materials and methods:**

The study was conducted in 2021 within the French association Silver Geek, which offers digital workshops using Nintendo Wii consoles to older people living in retirement homes and organizes annual amateur competitions between teams of older adults. This qualitative study involved 16 participants (eight women, eight men) aged 63 to 97 years. Semi-structured interviews were used to explore the motivations, emotional well-being, and social impact of esport on older people.

**Results:**

The results show that older people who participate in esport are motivated by the discovery and mastery of new game skills, as well as the pleasure of performance through social comparison. All experience a psychological well-being that translates into intense positive emotions. Moreover, the esport context, whether during the pre-competition workshops or the competitions themselves, proved to be a strong vector for positive social relationships, especially with the young e-sports coaches. However, this activity had little impact on existing family relationships.

**Conclusion:**

This study highlights the potential of esport as a recreational activity to promote mental health and social integration in older adults. The challenge for future research is to identify the levers that will enable this activity to become a true medium for intergenerational communication.

## 1 Introduction

According to a report by GlobalWebIndex (2020), the number of gamers aged 55–64 worldwide has increased by 32% since 2018. In France, the Syndicat des Editeurs de Logiciels de Loisirs (2023) reports that all generations participate in video games, with an almost equal split between men and women. Almost half of people aged 65 and over (47%) play video games, while this figure rises to 61% for those aged 50–64 years. This increase can be explained not only by the widespread use of digital technologies, but also by the fact that older generations have grown up with video games and have continued to play them over the years. Most have remained active gamers. These data show that older people, whom we define here as people aged 60 years and over, in line with the World Health Organization (WHO, 2016) definition, must now be considered as players in their own right in the world of video games.

Video games are now considered a sport practice in their own right, involving the cognitive (attention, concentration, etc.) and physical (dexterity, coordination, rapid reflexes, visual acuity, etc.) abilities of individuals, alone or in teams, by electronic media (Hallmann and Giel, 2018). As a result, the scientific community was quick to focus on the benefits of these video game practices for the health and quality of life of older people (Steinkuehler, 2020).

Gerontological approaches consider video games primarily for their qualities of being utilitarian (e.g., maintaining health) and pragmatic (e.g., access to digital practices). From this perspective, exergames—active video games that associate physical movement, strength, balance, and flexibility with cognitive abilities—can alleviate the lack of physical activity among older people (Boj et al., 2018; Zeng et al., 2017) and improve their cognitive health (Maillot et al., 2012a; Maillot et al., 2012b). Playing exergames also has a positive effect on mood (Onishi et al., 2022), reduces anxiety (Viana et al., 2020), and improves the psychological well-being of older people (Chao et al., 2015). The social well-being that video games provide for older people has also been perceived as an end in itself (De Schutter, 2011; De Schutter and Abeele, 2015; Iversen, 2016; Osmanovic and Pecchioni, 2016). In general, training older people in new technologies such as iPads has been shown to contribute to their social well-being. By giving them access to online communities, older people can renew old relationships and improve communication with their families (Delello and McWhorter, 2017; Winstead et al., 2013).

As far as the benefits of video games in terms of sociability are concerned, the research performed by sociologist Lavenir (2023) led to a more mixed assessment. Based on in-depth interviews and observations of video game workshops, the author found that “video games are difficult to integrate into the social life of older people,” a social life that becomes increasingly fragmented as people age and move into institutions. Nevertheless, the author noted that the workshops offered in these institutions for the old (medical homes, independent housing, and community centers) are one of the few places where various forms of social interactions around video games can develop: exchanges between participants as well as those with the young volunteers responsible for the activities and the staff of the host institutions and associations that promote these activities. However, as Lavenir indicated, this promotion of access to digital technology and video games for older people, this desire to “bridge the digital divide” between generations, can be seen as a part of a social injunction to “age well” in a way—here, through a useful leisure activity—that does not appeal to older people (Puijalon and Trincaz, 2014).

As we have seen, the impact of video games on the health and quality of life of older people is now well documented. However, it appears that no research has yet specifically addressed the psychological challenges associated with older adults’ participation in esport competitions (Kelly and Leung, 2021; Onishi et al., 2022), particularly those living in retirement homes. Esport, or electronic sport, are defined as “codified confrontations between players via intermediate screens during national and international video game competitions” (Besombes et al., 2016). Professional esport organizations are emerging around the world, with teams and leagues, professional players, coaches, analysts, and commentators. Esport competitions are often broadcast live on online platforms such as Twitch and YouTube Gaming, and are the reason for major gatherings such as the Southeast Asian Games and Paris Games Week. Esport have traditionally been played by young players who have been trained in gaming from an early age. The inclusion of older players in the world of gaming, including esport, is more recent, but is growing rapidly, leading to the emergence of specific categories for them at major esport events.

The specific psychological challenges faced by older people in the world of esport are numerous and raise many questions: Beyond the institutional expectations of esport, what are the stated motivations of the participants? Can competition increase the psychological well-being of older people through positive emotions? In contrast, can the inability to progress, loss of skills, or poor performance in competition confront older adults with the decline of their own physical and mental abilities, leading to lower self-esteem and social withdrawal? Are esport a way for older people to enrich their social relationships, especially with their families? The specific objectives of this qualitative study were guided by three research questions that provided a framework for providing answers to these questions.

*Research question 1: What motivates older people to get involved in esport?*

Several studies have focused on the problem of motivation among older adults engaged in video game practices. Some older adults are primarily motivated by the search for well-being, the maintenance of skills, or the fight against boredom (Hall and Marston, 2015; Goldstein et al., 1997; Farris et al., 1995). For others, the main motivations for playing included pleasure and relaxation (De Schutter, 2011), an escape from reality (Pearce, 2008; Nap et al., 2009), and an opportunity to maintain social relationships (Schultheiss, 2012), especially by playing video games with family members. In addition, De Schutter (2011) highlighted the challenge—the desire to progress. Nevertheless, as far as we know, no study has looked specifically at the motivations of older esports players.

Esport are not just the recreational practice of video games; it creates a context that may favor processes of social comparison at different levels: self-comparison of one’s own performance over time, and comparison with other more or less older adults during competition.

In this context, the motivation of older adults can be examined through the lens of the achievement goal model (Wolfe and Crocker, 2003), which was recently updated by Urdan and Kaplan (2020). This theoretical framework is widely used to understand how personal goals impact behavior and performance across various contexts, including work environments, education, sports, and, more recently, esport (Svensson, 2024). According to this model, individuals can pursue two types of goals either independently or in combination. Performance goals emphasize the “ego,” or the pursuit of self-worth through social comparison; in esport, these might involve winning matches and advancing in rankings. In contrast, mastery or learning goals focus on the “task,” which involves understanding or mastering new skills; in esport, these goals might target improving precision or skillful gameplay. To date, few studies have applied the achievement goal model to explore the motivations of older adults to engage in physical activities and the effects of these motivations on their physical and psychological well-being. Notably, Riou (2014) demonstrated greater instability in self-esteem among older adults compared to younger adults (Study 1) and highlighted the benefits of engaging in physical activity within a mastery-oriented motivational climate for very old and institutionalized individuals (Study 5). However, no research has yet investigated the link between older adults’ motivations for playing video games and their well-being. Building on Riou et al.’s (2012) study of older adults in specialized care facilities who participated in physical activity sessions, we hypothesize that a motivational climate that emphasizes the discovery or mastery of new skills (mastery or learning goals) could lead to adaptive strategies in response to the challenges associated with aging and declining abilities. Therefore, the psychological well-being of older adults participating in esport may be related to well-managed strategies for pursuing both mastery and performance goals.

*Research question 2: What are the potential effects of esport on the emotional well-being of older people?*

Emotions are an integral part of video game play. Several studies have shown a causal relationship between playing video games and increased positive emotions and mood (Russionello et al., 2009; Ryan et al., 2006). According to Granic et al. (2014), the context of video games can lead to the control and modulation of negative emotions. In other words, playing video games helps inhibit certain negative emotions at the expense of more positive ones. The enjoyment of video games is an important factor, as it depends on the player’s ability to satisfy psychological needs, such as a sense of competence and autonomy (Tamborini et al., 2010). With regard to video game practice for older people, Jung et al. (2009) showed through a six-week intervention on the use of Nintendo Wii with 46 residents aged between 56 and 92 years that residents who played Nintendo Wii experienced greater emotional well-being compared with residents who played traditional games. With respect to esport, Behnke et al. (2022) deliberately activated different emotions (anger, enthusiasm, sadness, and amusement) through several film scenes. Their aim was to test how emotions affect their performance in an esport video game (e.g., FIFA 19). Enthusiasm and fun led to better scores, mediated by a tendency to approach performance related to goal chasing. However, given the paucity of studies on this topic, the aim of this study is to identify the adaptive emotion regulation strategies that contribute to the well-being of older people involved in esport.

*Research question 3: What influence does esport practice have on older people’ social interactions?*

As physical proximity is no longer a necessity, online gaming provides older people with the opportunity to maintain intergenerational relationships (Osmanovic and Pecchioni, 2016, 2017). Lavenir (2023) examined the contribution of video games to the social relationships of older people. In the players’ accounts, the author noted that few of them mentioned having asked for or received help from friends or family to solve a problem or do something new in video games. Among older people, social networks are organized around friends and neighbors, a few privileged horizontal family ties (siblings, cousins, brothers-in-law, and sisters-in-law), and adult children (Desquesnes et al., 2018). Given the media coverage of video games, we can assume that the competitive practices of older people appeal to their family network, and more specifically to their youngest offspring (grandchildren, nephews, nieces, etc.). This study therefore analyzed the family intergenerational impact of esport among the elderly.

## 2 Materials and methods

### 2.1 Study design

This empirical study was conducted using a descriptive qualitative methodology, which is often used in healthcare (Kim et al., 2017).We chose this methodology because it met our exploratory research objectives: to observe and describe the e-sport experience as accurately and faithfully as possible, as perceived by the participants. It was suited to the objectives sought: to analyze a specific activity (esport) within a target population (older people living in retirement homes) which has been the subject of little research to date. Semi-structured interviews were conducted, guided by the three main research questions, and the data were processed through thematic analysis to generate meaningful interpretations.

### 2.2 Participants and procedure

The study was conducted in 2021 within the French association Silver Geek, which fights against “illectronisme” (digital illiteracy) by offering older people living in retirement homes with digital workshops using tablets and Nintendo Wii consoles. The association also organizes annual amateur competitions between teams of older adults, the Silver Geek Senior Trophy, whose regional finals take place at major events, such as Gamers Assembly, Paris Games Week, Japan Expo, and Stunfest. In some of the retirement homes where the Silver Geek association is active, volunteer residents prepare for the competitions by taking part in weekly Wi-Bowling workshops run by young coaches aged between 18 and 25 years.

A list was made of older people who had participated in one or more of these esport competitions in the previous 2 years. The people on this list were then contacted through the management of their homes, who had been informed of the aims and procedures of the study. Of these people, only those who had given their consent to the management of their establishment were offered an appointment to be interviewed by one of the four members of the research team (CE, RG, CB, or LD). On the day of the appointment at the retirement home, their consent was sought again.

The location of the interview was left to the discretion of the interviewees, who were free to choose a quiet and familiar place. At the beginning of each interview, information on the study was presented. We explained to the older people that the interview would be recorded and promised to keep their personal details and information confidential, strictly protecting their privacy. Written informed consent was obtained thereafter.

Finally, the sample consisted of 16 participants, eight women and eight men aged between 63 and 97 years at the time of data collection, who were found in eight retirement homes. Five of these participants (the youngest, living in residences B and H) were cognitively impaired. The sample characteristics are shown in Table 1.

**Table 1:** Characteristics of the competitor sample.

Participant	Sexe	Age	Residence	Family situation
SB	M	89	Residence A	Divorced, 3 children
RV	M	79	Residence A	5 children
EM	F	93	Residence A	Widow, 5 children, 11 grandchildren, 11 great-grandchildren
SM	F	93	Residence A	Widow, 2 children, 4 grandchildren, 5 great-grandchildren
JLN	M	67	Residence B	Celibate without children
RP	M	64	Residence B	Celibate without children
FV	M	67	Residence B	Celibate without children
MB	M	80	Residence C	Married, 3 children, 5 grandchildren
AB	F	87	Residence D	Widow, 3 children, 3 grandchildren
DB	F	93	Residence E	Widow, 3 children, 11 grandchildren
BX	M	97	Residence E	Widow, 1 children, 2 grandchildren
JM	F	90	Residence F	Widow, 4 children, 9 grandchildren
LF	F	94	Residence F	Widow, 2 children, 3 grandchildren
CO	F	92	Residence G	Widow, 2 children, 3 grandchildren
GA	F	63	Residence H	Celibate without children
AD	M	69	Residence H	Celibate without children

### 2.3 Instrument

A semi-structured interview guide was developed through literature retrieval, research group discussion, and pre-interview revisions. Socio-biographical questions regarding age, family, leisure activities, and video game habits were asked, followed by a sequence of questions: (1) Can you tell me about your social relationships, usually? (2) How do you feel about playing video games, usually? (3) What motivates you to play video games competitively? (4) Have you ever played with your family (children, grandchildren, and others)? If so, tell us about a time when you played together? (5) Do they know that you are a competitor? If so, what do they suppose? Do they participate in these competitions? What kind of relationship do you have with them during the competition? (6) Would you like to share a memory related to these meetings? Do you have anything to add?

The questions were adapted to each person’s level of verbal comprehension and expression. The guide was therefore used as a framework around which the questions could be adapted and reformulated until it was certain that what was being said was correctly understood. Inducement and suggestions were avoided. Each interview lasted for 20–40 min.

### 2.4 Data analysis

The recorded conversations were transcribed verbatim. The transcripts were then individually verified against the original recordings to ensure accuracy. Inductive thematic analysis was used to analyze the data manually, following the procedures suggested by Braun and Clarke (2006): (1) Two researchers, CE and RG, read the transcripts independently to familiarize themselves with the data and then extracted information on the research questions separately. Information that was not directly related to one of the research questions but seemed relevant was also retained. (2) The whole research team then proceeded to generate codes manually. The four researchers independently read all verbatim transcripts and assigned codes to the significant dialogs. (3) By grouping these codes into units of analysis, it was possible to identify the common themes raised in the interviews and associated with the research questions. In subsequent face-to-face meetings, the research team reviewed and verified the themes and split, merged, or deleted certain codes. Any differences in opinions about the themes and codes associated with them were discussed together to reach a consensus. A total of 15 codes were identified and divided into five themes (Table 2). The occurrences of each participant on each of these codes were counted (present one/absent zero), and the transcripts associated with these codes were used to interpret the data.

**Table 2:** Themes, codes and number of occurrences.

Themes	Codes	Number of occurrences
Motivations	Discover, progress	12
	Win, do better than the others	11
	Enjoy the atmosphere	6
	Keep busy	1
	Play	1
Emotions	Joy, pleasure, happiness	16
	Annoyance, nervousness, frustration	4
Perceived social links	Social link with family	10
	Social link with residents	5
	Social link with friends	3
Social links around Esport	Social link with young coaches	10
	Links with residents	3
Cross-generational social links around video game and Esport	Exchanges with family about video game	14
	Play video game with family	2
	Use of digital tools for communication	2

## 3 Results

To present the data, the most representative transcriptions linked to each theme and code are displayed below.

*Research question 1: Older people’s motivations for taking part in esport.*

In terms of “Motivations,” the intentions of ‘discovery and progress’ related to mastery goals were expressed by 12 participants/16. For example, we have heard “*My goal is to progress as much as possible” (FV), “I had to learn everything because it was the first time I’d played with a tablet. We’re getting by now” (JLN), “It’s something I’d never done before. I did not think I could do it. At first, I wanted to stop. And then, well, it’s gone and there it is. There are times when you surprise yourself” (DN), “There’s a movement you have to learn for bowling. I try to understand, especially what’s going wrong” (RV)* or even *“well, you have to try... you try to correct yourself” (CO).*

Almost as many participants (11/16) expressed performance goals in terms of ‘winning/being better than others’. So, for example, we noted” *You go there to win, but it’s not something that bothered me. You won, it was good; you did not win, too bad. There were people who played well; they won first place. It was very good for them; I would have preferred us to win, but it did not really matter (EM),* “*I’m happy if I get a good score. I do not really play to be first, not that I would not like to, but as far as possible not to be last” (SM), “Normally, the aim is to win. When I can win, I’m happy” (JLN) or even “This year, we have got a chance and we are going to do everything we can to make it happen. We do not know what the outcome will be, but we do not want to fail” (MB).*

Of these participants, nine expressed mastery and performance goals combined, for example: “*If you are playing something, generally the aim is to win. But, it’s not always easy to be in first place, so if you do well, so much the better. A man’s aim is to achieve the best he can, to do the best he can. The main thing is to get there, but the best rank is even better, preferable even” (RV), “In my life I’ve always tried to find something I do not know. I like discovering things, but if you play, it’s to win” (BX).*

Although it was not their only motivation, six participants were motivated by ‘enjoy the atmosphere’, for example: “*What I’m looking for is the atmosphere; it’s encouraging, it motivates, you think it’s annoying, but on the contrary, I enjoy it” (RV),* “*I like the atmosphere, there are so many people there for us”(AD), “We liked it, when we met in the corridors, good to go, on such a day, let us do it again! There you go. Well yes, we were waiting for the day”(CO).*

One participant said that their only motivation was to ‘keep busy’ in these terms *“It’s just to pass the time, and it’s because someone’s coming to get me”(LF)* and one participant suggested that ‘playing’ was an end in itself: *“Bowling’s the same; if it does not fall, too bad, as long as I’m playing, and that’s that. I’m not looking to win. I just enjoy playing and that’s that. I like games, all games”(AB).*

*Research question 2: The effects of esport on older people’s emotional wellbeing.*

In terms of “emotions,” all participants expressed a feeling of emotional well-being in esport practice through positive emotions such as ‘joy, pleasure, cheerfulness’ associated with emulating the collective game and the competitive atmosphere. For example, we have heard *“The weather was superb, people were cheerful. I was with a young and enthusiastic companion. I had a good day. That’s all there was to it” (BX), “We enjoyed it. Oh yes, when we met up sometimes in the corridors, we’d say to each other, “Come on, let us do it again!” We were waiting for the day” (CO), “So there are several tracks, so if one of them wins, the others watch and encourage them. To play in this, you have to be good. Ah, it’s a crazy thing” (RV)* or even *“To see people so enthusiastic, so involved, so supportive of the person who was playing. Oh that amused me, that’s what it was all about. It was nice. We got a lot of applause. People were shouting, people were applauding, as if they’d always known me (laughs)” (EM), “Well, it was hot, wasn’t it? (laughs), because it was close. We did not win by much. It was crowded. Ah, yes, it was impressive, in fact at one point, we completely lost concentration because of the audience saying “Go, go” (laughs), so that completely took our focus off. It’s an emotion, it’s stage fright. Good memories” (JLN).*

Four participants expressed ‘annoyance and nervousness’ about the game or frustration after a defeat, with a sporting attitude: *“To play three times, you have to wait an hour because the lady (mimes) gets up and does not take the ball. So we show them, we explain to them how to throw: either they are too close or they are too far away”(RV), “I do not like losing. No, it does not get on my nerves but, er, it gets on my nerves when you see the other person overtaking you. Ah, it’s irritating” (BX),*“*We were eliminated at the regional level, which was upsetting. We were penalized because we’d never played automatic and did not know how to do it. It’s another way of playing, because when you have the controller, you cannot touch the buttons, you cannot aim. As soon as you make a move, the ball is gone. So that’s what upset us a bit” (MB) and “Frustrated. Well, of course, we’d always like to win, but the others want to win too”(DB).*

*Research question 3: The influence of esport practice on older people’s social interactions.*

The question on the participants’ social relationships was approached from three themes: the “perceived social links” at the time of the interview, the “social links around esport” and the “cross-generational social links around video game and Esport”.

In terms of perceived social links, 10 participants mentioned their ‘relationship with their family’. Of these, seven participants reported a feeling of positive social support associated with a sense of well-being (for example, “*I have grandchildren who are adults, but there are also teenagers who are still studying. Of course, I’m very interested in what they are doing. I have the same children every day on the phone or on video. My children are my help” (EM), “My son phones me at least every other day to see how things are going, what you are doing” (CO), “My daughter calls me. My son does not call me as much, but he calls me all the same. So, if I need him, he’ll be the first to come, so I cannot complain*”). Three others expressed a feeling of distance from their family (“*My sister and I do not talk much, we each live our own lives” (JLN), “Family? No, never any news, unless they send me a greeting card, and they sent me a card for my birthday. Everyone here is an individual, there’s selfishness here” (RV), “I still have family but I do not see them very much” (RP).*

Five participants spoke about their ‘relationships with other residents’, including four in a positive way and one in a negative way, as “*I feel good with everyone here” (SM)* or *“Here, everyone is in individual. So there’s a selfishness” (RV).* Moreover, three participants spoke of maintaining communication with ‘friends outside the resident’ as “*I have friends with whom I correspond by email but there are people who have disappeared. Otherwise I’ll call them. The telephone is a great help” (EM)* and “*There’s the president of my village’s festival committee, for example. Then there are other people, and a lot of people around me” (MB).*

Regarding the “social links formed around the esport” practice, three participants spoke about their ‘social links with other residents’ during the bowling workshops in positive terms. For example: *“Bowling brought us closer together, and we became really good friends with R. What was great about R was that he was into strikes and I was into spares” (JLN)* and *“At the residence, we are a small team of bowling friends” (EM).*

In the same context, 10 participants described the richness of the ‘social links with the young coaches’ who run the workshops. For example: “*I play with 2–3 people and with the small team of young people who run the workshops. They come to train us and give us advice. It’s very pleasant because they are between 15 and 18/19 years old. It goes well; you can talk to them, we can exchange ideas; they are very nice, they are interested in older people, and it’s mutual (EM),* “*I took their name. It’s great and I’ve always liked young people. So, it’s all the easier to adapt to them” (MB),* “*There was good contact; they did not consider our disabilities, and they took us for normal people. We made friends with these young people” (SM); “He′s very nice, they were two for a while and now he’s on his own but he’s very nice”(AB)* or even “*we got on really well with the young people, they’d say, “Come on, Grandpa, (laughs), come on, Grandpa, let us go!” (BX).*

Regarding the “cross-generational social links around video game and Esport,” of the 14 participants who said they ‘exchanged with their families about video games and esport’, eight said they had heard their families express pride and six said their families were relatively indifferent. For example*: We participated in the competition and then I had the grandchildren. The youngest, Paul, who’s about to turn 16, came with another friend, and then my son, the friends; there were about 30 of them cheering me on (MB),* “*They were in awe of their grandfather. When we came back, ah, the cousins, even cousins from far away, said, “Hey B, you are a champion!”(BX),*“*They’re happy with me. They said, “The brother’s not joking, he won. R came second in Paris. Oh yes, the brother’s not doing badly.” (RP)*, *“At the end of the tournament, when the results came in, I got some phone calls, or at least a text message, from my grandchildren, saying “Congratulations!” It was my granddaughter who sent them a photo” (SM),* “*I spoke to my children about it. My son said to me, “Mom, keep as busy as you can.” (LF)* or even *“Well, they say it’s good for you. I’m the one who told them, so they are not too surprised” (GA).*

Two participants said they sometimes ‘played video games with their families’, as “*Yes, once, but it’s not very often, because I have to go to their house. There has to be a computer, a TV screen,* etc. *So, it does not happen often” (EM) and “My grandson plays and then he tells me it’s better if you do that, and that’s that. We talk about how to play, but it’s not easy to play together over the internet” (BX)*, and two said they ‘used digital tools (tablet, computer) to communicate’ with their families as *“I exchange text messages with my grandchildren” (SM).*

## 4 Discussion and implications

Analyzing the feedback from older people who have taken up esport provides several insights into our research questions. None of these people expressed a motivation related to maintaining their cognitive or physical abilities, and very few were simply looking to keep busy. Instead, esport appears to be more than just a game; it serves as a platform for deep engagement. In line with the achievement goal model, both mastery (discovery and progress) and performance (winning and outperforming others) goals were spontaneously expressed by participants. This suggests that the competitive environment of esport encourages the development of achievement goals, in line with Wolfe and Crocker’s (2003) model.

Furthermore, in line with Jung et al.’s (2009) results, the interviews revealed that esport contributes to the well-being of these older players. They reported experiencing intense positive emotions, such as joy, pleasure and good humor, associated with the collective excitement of competition. In fact, the competitive atmosphere seems to be an important motivating factor for many participants. While a few participants mentioned negative emotions such as nervousness and frustration, these were typically associated with specific competitive contexts. None of the participants mentioned being permanently upset as a result of too much pressure or failure during a competition.

In terms of social relationships, esport seems to be a source of positive social experiences for these very older adults. For more than half of the residents, the esport environment - whether during the preparation workshops or the actual competitions - proved to be an important source of positive social interactions, especially with the young esport coaches. As evoked by the participants, this connection with the young coaches around the video game and the competition refers to the restoration of a welcome intergenerational link. This connection also extended to the family members of some participants. Notably, half of the participants reported receiving expressions of interest and pride from their families. Such positive reinforcement, particularly from grandchildren as highlighted by several participants, suggests that esport can serve as a bridge between generations, fostering meaningful interactions. However, these interactions remain limited outside of competitions, as only two participants had occasionally played video games with family members.

Moreover, some participants noted their families’ indifference toward their involvement in esport. The accounts we gathered do not clarify whether this lack of engagement pertains specifically to esport or reflects a broader disinterest in any activities undertaken by their elderly in the retirement home. In any case, our findings raise important questions about the factors that shape family support, including generational attitudes toward technology and gaming culture.The potential for video games to become a new medium for intergenerational communication likely requires addressing certain stereotypes and prejudices surrounding older gamers. A shift in societal perceptions of older individuals participating in esport is also necessary. To foster this shift, families could be more actively involved in workshops or preparations for competitions, helping bridge the gap. Additionally, there is a need for greater recognition and inclusion of older players within the esport community.

In conclusion, the esport program offered in retirement homes was well received by participants. Both during training and in competitions, they found a sense of fulfillment through the joy of discovery, the acquisition of new skills, and competitive play, without their psychological well-being being adversely affected by failure. These findings align with the perspective outlined by De Schutter and Abeele (2015) in their ‘gerontoludic manifesto,’ which emphasizes the playful and hedonic aspects of activities for older adults. The esport workshops provided by the Silver Geek association do not aim to address gerontological issues such as preventing or rehabilitating cognitive and physical decline. Instead, participants perceived these workshops purely as a recreational activity. While it is undeniable that aging involves some degree of decline and weakening, none of the older individuals expressed concerns about meeting esport expectations.

This study has several limitations that should be acknowledged to inform future research directions. The primary limitation is the small sample size, which restricts the ability to generalize the thematic frequencies observed to a broader population. The sample included individuals of varying ages, cognitive levels, and verbal expression abilities. Specifically, five participants (from residences B and H) had cognitive impairments and minimal family connections (e.g., single, childless). This variability may affect the reliability of the findings, as not all participants had the same capacity to articulate their esport experiences. Nonetheless, this heterogeneity underscores the diverse experiences of older individuals engaging in the same activity. Previous research has emphasized the importance of recognizing that older gamers are not a homogeneous group. Accounting for factors such as age, gender, and psychosocial context can provide a more nuanced understanding of motivations and the diverse meanings associated with video games (De Schutter and Malliet, 2014; De Schutter & Van den Abeele, Harley et al., 2010; Harley et al. Harley et al., 2010).

## 5 Conclusion

Integrating older people into the digital age presents a significant social challenge in terms of public health. Increasingly, the video game industry, the medical field, and organizations dedicated to promoting “healthy aging” are striving to create opportunities for older adults within the gaming world. Research over the past decade has demonstrated that regular video game play can help mitigate certain forms of cognitive and physical decline, develop digital skills, and preserve social connections that often diminish with age. Despite these findings, research specifically on esport for older adults is still emerging. Our study highlights the potential psychological and psychosocial benefits that older individuals may gain from participating in esport competitions. However, further research is needed to identify who is most likely to benefit, assess potential risks, and explore strategies to mitigate these risks. The competitive environment of esport appears to positively impact psychological well-being. To ensure these benefits are sustainable and not limited to isolated events, it is crucial to explore how to foster intergenerational connections. This would help esport become a meaningful venue for sharing both personal and collective experiences across generations.

## Data Availability

The raw data supporting the conclusions of this article will be made available by the authors, without undue reservation.
